# CFRV: A Decentralized Control-Flow Attestation Schema Using Mutual Secret Sharing

**DOI:** 10.3390/s22166044

**Published:** 2022-08-12

**Authors:** Yuanpei Li, Qinglei Zhou, Bin Li, Yan Zhuang

**Affiliations:** 1 School of Cyber Science and Engineering, Zhengzhou University, Zhengzhou 450001, China; 2 School of Computer and Artificial Intelligence, Zhengzhou University, Zhengzhou 450001, China

**Keywords:** internet of things, software integrity, control-flow attestation, challenge–response

## Abstract

Control-flow attestation (CFA) is a mechanism that securely logs software execution paths running on remote devices. It can detect whether a device is being control-flow hijacked by launching a challenge–response process. In the growing landscape of the Internet of Things, more and more peer devices need to communicate to share sensed data and conduct inter-operations without the involvement of a trusted center. Toward the scalability of CFA mechanisms and mitigating the single-point failure, it is important to design a decentralized CFA schema. This paper proposed a decentralized schema (CFRV) to verify the control flow on remote devices. Moreover, it introduces a token (asymmetric secret slices) into peer devices to make the attestation process mutual. In this case, CFRV can mitigate a particular kind of man-in-the-middle attack called *response defraud*. We built our prototype toolbox on Raspberry-Pi to formulate our proof of concept. In our evaluation, CFRV protects the verification process from malicious verifiers and the man-in-the-middle attack. The proposed mechanism can also limit the PKI (Public Key Infrastructure) usage to a single stage to save the peer devices’ computational cost. Compared to related decentralized schemes, the cryptographic operation’s duration is reduced by 40%.

## 1. Introduction

The Internet of Things (IoT), mainly consisting of intercommunicating embedded devices, is evolving rapidly in both scale and functionality. Its territory has expanded beyond the laboratory to cyber-space infrastructure and other industrial applications. Applications such as IIoT [1], V2X [2], edge computing [3], and smart city [4] have benefited from the network formed by this massive quantity of embedded devices. In this surge of IoT applications and services, devices depend on software to meet required functionality and flexibility. However, designing a vulnerability-free program with no implementation flaws in its life cycle is difficult. Moreover because of the limited computing resources of embedded devices, their applicable security mechanisms are also relatively limited. Thus, software running on IoT devices may introduce more pervasive and challenging security issues into the cyber-physical world in this inter-connection process.

Current software exploitation approaches have evolved from code injection to code-reuse (e.g., return-oriented programming [5]). Code-reuse attacks are an advanced exploitation technique that can bypass security mechanisms like data execution prevention (DEP) and code signing [6]. Through this approach, attackers can construct a chain of legitimate code snippets for malicious usage by redirecting the control flow. To cope with these attacks, researchers have developed several code-reuse mitigation mechanisms like control-flow integrity (CFI) [7], code-pointer integrity (CPI) [8], and control-flow attestation (CFA) [9].

In most cases, the control flow can be considered as the reflection of software behavior. CFA fills the gap between control flow integrity (CFI) and software remote attestation (RA). It extends this remote attestation schema from claiming the integrity of a static binary to to determining the validity of its run-time behavior. The key security feature of CFA is a secure control flow recording module. With hardware or software protection, this module can securely log control flow events during the execution of the software. Since these records cannot be tampered with without making physical damages [9], verifiers can obtain the proof to determine whether the software behavior was integrated through examining its records. CFA still has room for improvement in the security of its verification protocol, since it is a newly proposed mechanism. A remote attestation schema that uses a trusted center would risk single-point-failure [10].

To meet the needs of making inter-operations between peer devices, it is not enough for a remote attestation schema that could not work without a central node. Moreover, security assumption in these works—requiring a trusted center—are strict. Therefore, it is necessary to develop a decentralized control flow attestation schema. In developing a decentralized attestation schema, a node could be a verifier in one verification session while being a prover in another session. With the protection of CFA, each device in the network is able to confirm the validity of the computation result that its peer devices transfer. This ability makes this CFA schema become more suitable for protecting software integrity in the growing complexity of the IoT landscape. Without a trusted center that provides the validity of the attestation request itself, a mechanism that mitigates the malicious verifier is necessary while developing a decentralized CFA schema.

This paper develops a decentralized CFA schema called CFRV (control flow run-time mutual validation). In addition to securely recording the control flow, it proposes a secret slicing mechanism between peer devices to reach a mutual verification process. The prover could determine both the origin and validity of challenges. It protects the attestation process from a kind of MiTM attack called *response defraud*, launched by a malicious verifier. CFRV, our proposed schema, can also relieve the performance burden by limiting the public-key-usage in resource-constrained devices to a single and only crucial stage.

Our contribution is elaborated as follows:

- We patched the decentralized CFA with a secret slice mechanism. It makes the prover able to determine the real origin of challenges and achieve mutual verification between peer devices.
- The proposed secret slice mechanism in CFRV can also extend those schemes which use the execution of challenges as the proof-of-execution. In the decentralized schema that requires the prover to execute the challenges, it refuses illegal challenges that contain attack vectors from a malicious verifier.
- Limiting PKI usage to a single phase (the *registration phase*) reduces the computational burden of handling the attestation, especially for resource-constrained devices.

This paper is organized as follows: We describe the research questions for approaching a decentralized CFA schema in Section 3. From Section 4 to Section 5—the central part of this paper—we propose our solution. Section 4 presents an overview of our schema. Section 5 is the methodology details to solve the research questions. The implementation of CFRV and its safety evaluation are presented in Section 6 and Section 7. We conclude our paper with a summary of contributions, limitations, and future work in Section 8.

## 2. Background and Related Works

### 2.1. Control Flow Attestation

Control flow attestation is firstly proposed in C-FLAT, which verifies the CFI of remote devices [9]. It tags on each control flow with an identifier sequence to record execution paths. With hardware-based protection like Intel SGX [11,12] or ARM Trustzone [13], it provides a secure control flow recording module on every prover to ensure that the record of control flow could not be forged. It operates in a challenge–response pattern to make the verifier determine the validity of remote control flow through the records in response. The verifier sends a challenge, and the prover executes it. The tags are triggered to log its control flow while the target software executes challenges. In this way, a device can obtain the necessary proof to determine the control flow integrity of its peer. It extended remote attestation of software integrity from static binaries into its dynamic behavior with a record. Compared to those static schemes like MAGE [10], SEDA [14], and SANA [15], control-flow attestation is an advanced approach that proves the software integrity can be dynamically verified. This attestation schema could divide into centralized [9,16,17,18,19,20,21,22] and decentralized [23,24,25] approaches based on its security assumption.

Tiny-CFA [16] lowers the hardware requirements of CFA which is oriented to the low-end sensors. ScaRR [17] extend the CFA schema’s scalability, making the attestation suitable for complex tasks. Based on a probabilistic model, MGC-FA [18] balances the runtime efficiency and recording granularity of target software. LiteHAX [19] is designed to extend this attestation schema to defend against data-oriented programming (DOP) attacks. Lo-FAT [20], ATRIUM [21], and Liu et al. [22] leverages the existing processor features to overcome the performance overhead caused by the instrumentation of software. In our study, we focused on securing the verification process in these related schemes. The prover in C-FLAT [9] and Lo-FAT [20] gives its response without verifying the identity of challenges, using the premise that there is only one trusted verifier. Without an identification mechanism, the prover could be maliciously exploited by a device impersonating the “trusted” verifier. Tiny-CFA also mitigates this impersonation by containing a token in challenges.

However, the centralized schema is still vulnerable to the single-point failure [23]. A trusted center is mandatory in these centralized CFA schemes, which plays the role of the only verifier. Once the central node is unavailable, it affects the availability of CFA in the entire system. Not only that, but the requirement of a trusted center is hard to satisfy in some clustered IoT devices.

### 2.2. Decentralized CFA

To meet the needs of remote verification in various interconnected devices, it is not enough for an attestation schema that could not work without a central node. Koutroumpouchos et al. [23] decentralized the attestation and proposed a mutual verification schema (CFPA) in edge devices through the deployment of the public key. The device in this system could be either a verifier, prover, or both. Verifiers use its private key to sign challenges so that a prover can determine the identity of challenges. Hristozov et al. deploy a device identifier composition engine (DICE) to securely generate control-flow proof rather than using custom hardware extensions of the CPU architecture [24]. The verifier in their work is not required to be a central node. With the deployment of PKI to identify each device, this schema is suitable for the decentralized scenarios. Nevertheless, because of the use of a public key, they claimed that this scheme is vulnerable to a malicious verifier that launches the denial-of-service (DoS) attack. ARCADIS [25] is another control-flow attestation scheme oriented to distributed devices. It determines whether or not run-time attacks compromise the software of asynchronous, distributed IoT services. A verifier uses its public key to keep the confidentiality of challenge besides its digital signature. This way, ARCADIS protects the decentralized control flow attestation process from session hijack. This schema could be further developed to lower the computational cost of public-key usage on resource-constrained devices [26].

However, without a trusted center to provide the validity of the attestation request itself, a malicious verifier could threaten the verification process between peer devices; this applies especially for clusters in which every device is capable of launching a verification. As declared in [24], malicious requests may harm the availability of the attestation process. Moreover, the malicious node can launch a new verification process (to become a verifier) to get the legal response to bypass the attestation (which it receives). We call this vulnerability *response defraud*, which is described in Section 3.2. Moreover, the malicious verifier conducting the *response defraud* with no need to conduct the session hijack or steal another’s a private key (the impersonation). In this case, it is hard to mitigate this threat only by deploying the PKI. To our best knowledge, there is still a lack of effective CFA schemes which are equipped to mitigate this threat.

### 2.3. Executing Challenges as a PoX

Proof of eXecution (PoX) is a mechanism that ensures the target software is indeed executed during an attestation process. It aims to ensure all the control-flow records are generated with the timely execution of target software [27]. The PoX of target software can be realized by a hardware flag which is hard for an adversary to tamper with. Tiny-CFA introduces PoX to lower the hardware requirements in CFA.

Besides keeping the records integrated, this research indicates that it is also important to ensure that the control-flow record is actually triggered by current inputs. Under this condition, the other approach of PoX is to use software input as the challenge and let the prover execute it. In ScaRR [17] and Lo-FAT [20], the verifier initiates the CFA by sending prover a program input *S*. To be the other kind of PoX, the verifier in these schemes only accepts the response triggered by *S*. It is hard for the adversary to predict the control flow triggered by the next challenge. Therefore, this mechanism forces the prover to execute the challenges that the verifier sends. In this case, an attacker is prevented from replacing the input to trigger the un-hijacked control-flow, which would allow the attestation to be bypassed. In other words, without the PoX of input, the verifier may not be able to cover all the execution paths of a prover. Nevertheless, if we extend this kind of PoX into a decentralized scenario, its security insurance should be further developed.

In previous decentralized CFA research, deploying this kind of PoX (which regards software input as a challenge) is hard. This is because, in the verification process, the prover cannot determine the validity of the challenge. Once the verifier is no longer trustworthy, a malicious verifier could use an attack vector as the challenge. We call this threat a *poisoned challenge*, which is discussed in Section 3.1.

### 2.4. Research Gap

This paper formulates the root causes of these shortages in Section 3. Moreover, we elaborate on the research gap that CFRV is designed to fix.

- Issue 1: The existence of a center node may lead to single-point failure in the network. The whole cluster of devices is unavailable once the center node corrupts. For an attestation schema, it is not enough in its functionality as it could only work unidirectionally.
- Issue 2: In the decentralized schema, adversaries have chances to become a verifier. Due to the potential malicious behavior of the verifier, previous decentralized control flow attestation schemes cannot use the input as the PoX mechanism. Moreover, the security issue caused by a malicious verifier is hard to mitigate only by an encryption process. The adversary can bypass an attestation process by using a defrauded response from a new verification process it launches.
- Issue 3: Each node can initiate or respond to the verification in a decentralized schema. Under this condition, the frequency of PKI usage would increase significantly compared to the attestation launched only by the trusted center. Moreover, due to the need to mitigate impersonation in the decentralized schema, PKI usage is more sophisticated. Although public-key algorithms for resource-constrained devices have made great progress in the last two decades, high-frequency usage might still lead to performance bottlenecks. In decentralized CFA schemes, the computational cost of using the private key in every challenge–response process could be further optimized.

## 3. Research Questions

In decentralized verification schemes, especially CFA between peer devices, a node could not only be the prover but also act as the verifier. As we declared before, a trusted verifier is a strong security assumption in most cases. Therefore, verification stages where a malicious verifier is involved might develop new threats.

### 3.1. RQ1: Making the PoX Mechanism (That Requires the Execution of Challenges) Suitable for Decentralized CFA Schema

As introduced in Section 2.3, some schemes use the execution of challenges as the PoX. These schemes require a prover to execute the challenge to monitor the control flow it triggers. In a centralized CFA schema, this approach of PoX is reasonable because the verifier in their security assumptions is claimed to be a trusted center. However, this mechanism is hard to be deployed in the previous decentralized schema if the verifier is no longer trustworthy. In this part, we consider *poisoned challenges* from a malicious verifier to be the root cause of this barrier. In Section 5, it is mitigated by the proposed secret slice mechanism, which makes this kind of PoX suitable for the decentralized schema.

**Figure 1:**
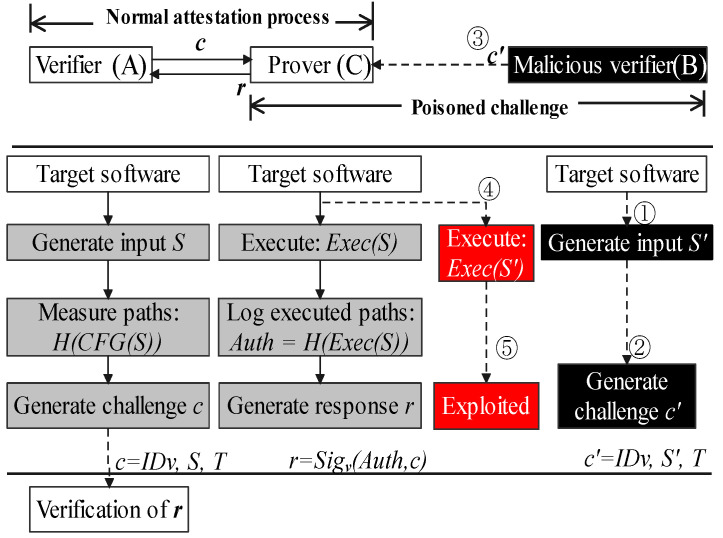
The execution of a poisoned challenge may enable the hijacking of a prover’s control-flow.

In other words, this paper regards the barrier that decentralized CFA schema cannot use the execution of challenges as the malicious verifier could hijack a victim’s control flow using the poisoned challenge. In Figure 1, verifier (*A*) and prover (*C*) are two innocent devices and running the same target software, acting as peer nodes. The gray squares indicate the normal attestation processes which are in line with previous works [17,20]. They use the execution of challenges as the PoX. The verifier (*A*) generates *S* as the input of target software. It records those execution paths that are triggered by *S* in the control-flow graph (CFG). Then, *A* generates challenge *c* by combining its verifier ID (*IDv*) and timestamp (*T*). Prover (*C*) executes *S* to show the corresponding control flow logs, and generates the response *r* which is a signed pair of challenge and execution paths, *Auth*. Verifier accepts the response, if *H(Exec(S))* equals to *H(CFG(S))* it stored, which indicates the prover has not been hijacked.

In the *poisoned challenge* process, a malicious verifier could exploit this execution process by injecting attack vectors as the payload in a challenge. After these poisoned challenges are executed, software running on the victim device could be under the control of adversaries for malicious usage. In Figure 1, *B* is a malicious device. ➀ uses return-oriented programming to generate attack vector *S’*. In ➁➂, malicious *B* launches a verification to *C* using exploit *S’* as a payload of challenge *c’*. The prover (*C*) has no efficient evidence to refuse this verification between peer devices. As a result ➃➄, shown in red squares, its control flow has been redirected after executing $S^{\prime }$.

### 3.2. RQ2: Mitigating the ‘Response Defraud’

Undoubtedly, the verifier could reveal adversaries in a device under an attestation process. This ability is the primary concern of CFA. However, in decentralized approaches, the malicious node has a further strategy (*response defraud*) to bypass the verification. It can launch an additional verification to defraud the legalized response from another device. This paper regards the *response defraud* as a particular kind of MiTM attack in the challenge–response scenario. However, the key difference between *response defraud* and the general MiTM attack is that the malicious verifier has no need for conducting the impersonation or session hijack. The main purpose of a malicious verifier is not to compromise software or steal the victim’s key but to defraud a legalized response in the new verification process. Each pair of attestation sessions is integrated and confidential. In this case, *response defraud* is hard to mitigate only by the PKI or pre-shared symmetric keys.

**Figure 2:**
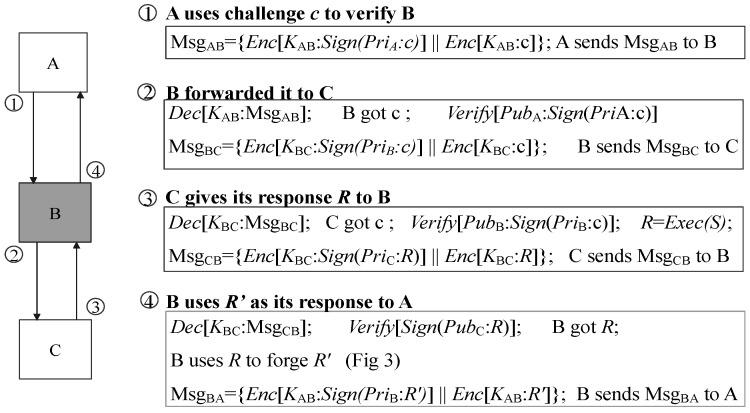
The response defraud in decentralized CFA schema.

In Figure 2, *A* and *C* are two innocent devices, while *B* is malicious, with its software control flow hijacked. In line with previous works [25], the verifier uses its private key to sign the challenge, and the verification session is protected by symmetric encryption. In ➀, *A* launches a remote verification to determine the control flow integrity of *B*. *B* can decrypt the message by using their session key $K_{AB}$. To defraud the legal response, in ➁, *B* signs the *challenge* using its own private key. After that, it launches the other verification to *C* with the same challenge. *C* is unaware of the process that *B* is being verified by *A*. In this case, it gives a response encrypted by session key $K_{BC}$. The malicious node decrypts the response using $K_{BC}$ and obtains a legalized response *R* from *C*. It re-encrypts it using $K_{AC}$. After the re-encryption, *B* sends it back to *A*. Due to the uncertainty of the network, it is hard for the verifier to set a strict time limit for verification duration. The malicious device could reach its goal once such a strategy is completed sooner than the session closed. As the device itself is malicious, the adversary owns the key stored on *B* during the communication.

**Figure 3:**
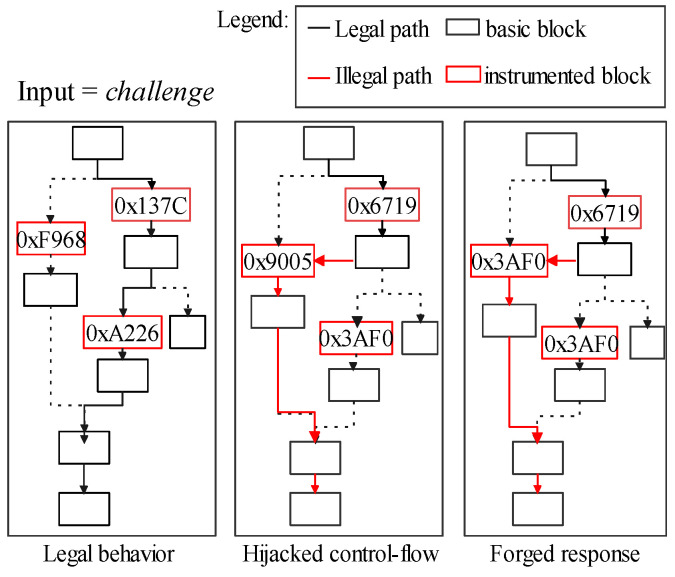
Forge a legalized response(R’) after the fraud.

Moreover, *response defraud* is still achievable for adversaries even if target software’s control-flow stamps (block-ids) are randomly generated. In Figure 3, *B* substitutes its control-flow stamps according to *R* to forge a legalized $R^{\prime }$. This is because those stamps, accompanied by the target software, are not directly protected by the CFA (i.e., the target software is stored in the *normal world*). Its implementation detail is in Section 7.1.4.

### 3.3. Motivations

In a decentralized attestation schema, each node could be a verifier in one verification session while being a prover in another session. Without a trusted center that provides the validity of the attestation request itself, a mechanism that mitigates the malicious verifier is required while developing a decentralized CFA schema. Therefore, to enhance the safety of the decentralized CFA process, CFRV tackles *poisoned challenges* (RQ1) and *response defraud* (RQ2) by patching the secret slice mechanism and its related support onto the control flow attestation in Section 5. It gives the evidence for a prover to determine the origin of challenges and their validity.

## 4. System Model

### 4.1. Overview

**Figure 4:**
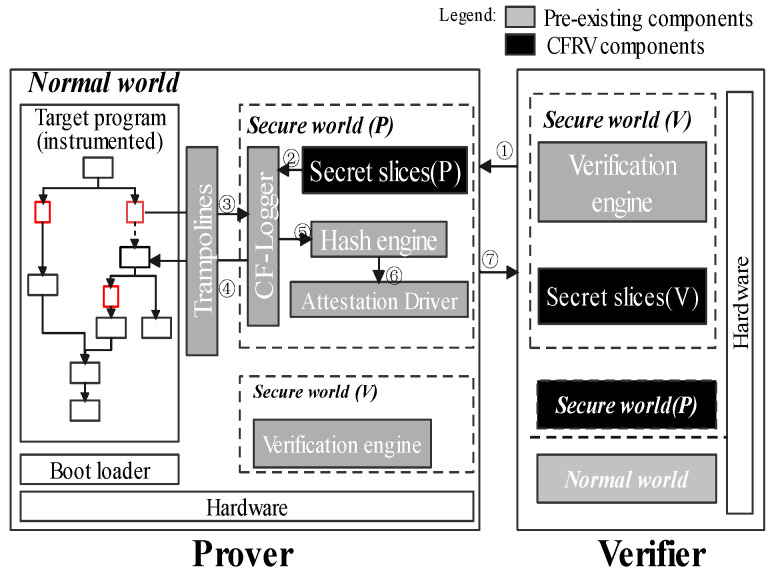
The remote verification architecture.

This section is mainly about the high-level description of CFRV. Figure 4 shows how a CFA mechanism records software execution paths with the help of *ARM Trustzone*. Gray-tagged components are in line with previous work, which used them to record the control-flow securely [9,16,20]. When the software is running in the prover, instrumented stamps in the control-flow are passed through the *trampolines* to the *secure world*, which are extracted by *CF-logger* to *hash-engine* (➂➃➄). Then, the records would be processed by the *attestation driver* to generate the response ➅. ➀ and ➆ are a classic challenge–response process. In CFRV, there is no dedicated prover or verifier. We logically distinguish between two peer devices in Figure 4 to emphasize their attestation detail. When a device becomes the verifier, the verification engine in its *secure world (V)* sends a challenge. The prover uses *secure world (P)* to give its response. Black-tagged components, the *secret slices*, can check the challenges they receive ➁. It makes the prover able to determine the validity of challenges and which device generated this challenge.

Its main idea is that one node (i.e., one device in the network) generates a set of challenges and distribute them secretly to other nodes. Furthermore, each node only shares part of the challenges they generated with other nodes, but no two other nodes hold the same challenge. When a node receives one verification request, it can immediately tell whether the verifier is legitimate by checking the challenge distribution history. This way, peer nodes are free from malicious verification requests. The attestation process requires the prover to execute the software input *S* that the verifier sends (to generate the PoX). In this case, CFRV is suitable for the task-splitting scenarios that require the remote device to provide a trustful computation result (e.g., making data that remote sensors collected trustworthy or confirming the validity of a *remote procedure call*).

**Figure 5:**
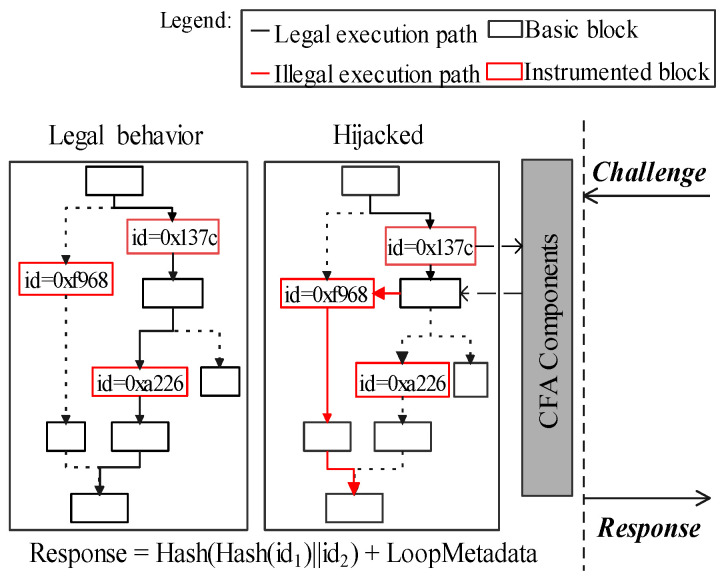
Attestation to a control-flow.

The mechanism which enables verifiers to ascertain the execution path of target software is in Figure 5. We stamp a message string in the control flow as the unique identifier for each execution path. These message strings are randomly generated during the compilation process. Once these instrumented stamps are being triggered, *CF-logger* in *secure world (P)* can record its execution path into a response. Thus, the run-time integrity of software can be determined by the occurrence order of basic blocks. An unexpected record would be considered a control-flow hijack. A verifier gives its judgments based on the pre-shared secret slices in its *secure world (V)*. Furthermore, we clarify the relationship between the secret slicer and other components in Figure Secretslice3-v2-part1 and Figure Secretslice3-v2-part2.

### 4.2. Security Assumptions

The adversary is capable of control-flow hijacking attacks in related work. Similarly, in our consideration, a control-flow hijack could be performed through code-injection and return-oriented programming. As the trust anchor, functions deployed in the *secure world* could not be disabled or modified as long as it has not been physically damaged. In addition, we make the security assumptions that there is no malicious node while they share secret slices. This requirement can be ensured by making devices share their secret slices only in the stage of device installation.

Moreover, we removed the requirement that the schema needs a trusted center to legalize challenges. Instead, we use a temporal master node that only operates in the *initialization phrase* once.

## 5. Mutual Verification Design

This section focuses on the working principle of *secret slicer*. It illustrates how to protect the CFA-based security mechanism from illegal challenges and malicious verifiers. We design the mechanism including the following steps: insert instrumentation in the control flow generates and then shares secret slices for mutual attestation. Thus, provers are able to confirm the validity of the verifier based on secret slices they hold. Therefore, peer devices could be protected from *poisoned challenges* and *response defraud*. Table 1 is the summary of the symbols and procedures used in this paper.

### 5.1. Design of Secret Slices

Secret slices are a key component of the verification process. To clearly illustrate its functionality, we present the principles of secret slices design in this part.

#### 5.1.1. Building Secret Slices

In order to reach a mutual verification, in our schema, the set of challenges used for verifying a particular device should be unique. In other words, every verifier has a unique challenge set, and they do not know about others’ challenge sets. We slice these challenge–response pairs to ensure that every challenge can only exist on one device. Similarly, we divide the challenge–response pairs (CRPs) to make sure that every response could only belong to one slice. This way, the prover can distinguish the one who generates the challenge while determining its validity.

We formalize these dis-jointed sets of challenges in Equation (1).

(1) $$\forall C_{i}\subset C,C_{i}\cap \left(C_{1}\cup C_{2}\cup \ldots \cup C_{n}\right)=\emptyset ;\;\forall R_{i}\subset R,R_{i}\cap \left(R_{1}\cup R_{2}\cup \ldots \cup R_{n}\right)=\emptyset$$

Moreover, we formalize its validity in Equation (2).

(2) $$\forall c_{j}\in C_{i},Exec\left(c_{j}\right)\in R_{i}$$

Note that we keep the functionality of every slice the same in Equation (3):

(3) $$\forall i,j\in n,R_{i}-R_{j}=\emptyset$$

**Figure 6:**
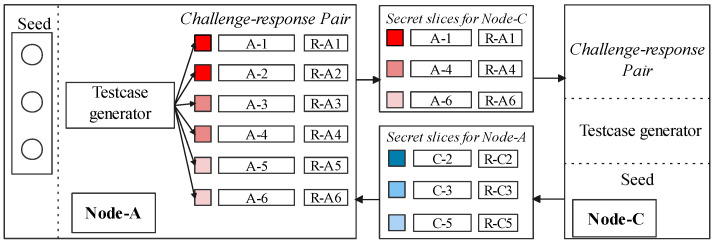
Building and sharing secret slices.

In Figure 6, we form the CRPs in the following steps: the secret slicer generates a set of test cases from the seed and records corresponding control-flow traces. The seed sets are used for automatically generating test cases and implementing one-time challenges the verifier keeps. The slicer makes target software execute these test cases to invoke instrumented instructions and to trigger the CF-Logger that records the control flow automatically. Then, *challenge–response pairs* are generated. Note that the control flow record is not the detailed trace of a software behavior but the result of hashing block-ids. Moreover, we assume the slicer has a seed set provided by developers to cover all the execution paths for the target software.

#### 5.1.2. Distributing Secret Slices

Each node—an embedded device in an IoT cluster—divides its CRPs into sets of disjoint slices based on the number of communication pairs. Figure 6 illustrates its operation between node *A* and *C*. The slicer delivers these secret slice of node *A* to other node *C*, as $Secret_{AC}$. Only the challenges can be stored in *A* after the delivery, and the responses in CRPs are removed. We define this challenge set as $challenge\_slice_{AC}$. The secret slice is a bunch of generated test cases and corresponding responses (e.g., $Secret_{AC}$). The slicer ensures that test cases make each secret slice able to cover all the execution paths. When sharing to some other nodes, e.g., *B*, test cases will be picked from another CRP division, which could also cover all the execution paths. This mechanism is guaranteed by the design of slicers. Moreover, a certain test case could and could only be picked once. This makes the content of a certain secret slice totally different from other slices. For example, test cases in $Secret_{BA}$ and $Secret_{BC}$ are different. In this way, the prover *A* can use the $challenges\_slice_{AC}$ to confirm that the challenge is actually from verifier node *C* when it requires the control-flow response of *A*. It is vice versa for $Secret_{CA}$ generated by verifier *C*.

#### 5.1.3. Safety

The secret slices can only be stored and used by *secure world (P)*, which the *Trustzone* protects. Challenges in the secret slices are only used for determining the validity of the prover. Target programs in the *normal world* (and adversaries) cannot obtain or execute those challenges stored in secret slices only when *secure world (P)* passes the challenge to *normal world*. During the secret delivery phase, secret slices are protected by public-key infrastructure (PKI). The malicious node cannot acquire others’ secret slices. In this way, we protected the response of control-flow of *A* from leaking to adversaries in the prover. Therefore, it enables the prover to distinguish masquerade challenges from a malicious verifier, which tries to impersonate some other legal ones. In our schema, the secret delivery works along with PKI, which is elaborated in Section 5.3.

### 5.2. The Mutual Verification

**Figure 7:**
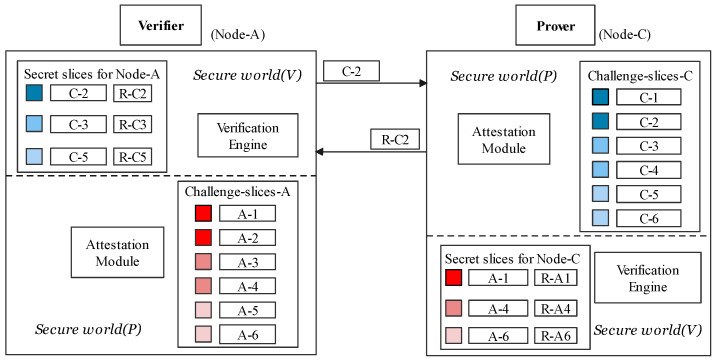
Mutual authentication using secret slices.

The verification process allows one node, which acts as a verifier, to test control-flow integrity on the other node randomly. The verification phase, as shown in Figure 7, consists of three following steps:

(i) The verifier *A* uses a random number $r_{i}$ to pick a *Challenge–Response Pair (CRP)*$\left[c_{2},R_{2}\right]=Secret_{CA}\left[r_{i}\right]$ from a secret slice sent by prover as $c_{2}$. Then, it sends $c_{2}$ as an attestation request to node *C*.
(ii) The prover ensures that $c_{2}$ is actually from $Secret_{CA}$ by confirming $c_{2}$ has actually been shared to *A* in the secret-sharing stage. The prover executes the challenge $c_{2}$ after the admission that it is from $Secret_{CA}$. $R_{2}^{\prime }$ represents the hash of basic-block id consequences of its execution path, which would then be sent to the verifier (V).
(iii) If $R_{2}^{\prime }$ is equal to $R_{2}$ that the verifier stores, verifier (V) would confirm the prover’s integrity based on its control-flow that matches the challenge in $Secret_{CA}$. The challenge is only in $Secret_{CA}$ which was delivered uniquely from *C* to *A*. Therefore, the validity of verifier *A* is also confirmed.

In this way, the prover can mitigate the *poisoned challenge* by checking if the challenge existed in the secret slice. Secret slices in *secure world (P)* refuse to pass such a poisoned challenge to followed components. The *response defraud* can also be extinguished by step (ii), where the malicious node could be revealed when it forwards the challenge to other devices; this is because the forwarded challenge has not been shared in secret slices. For example, *A* could not use $c_{1}$ to verify *C*. If it is used, the prover *C* would refuse the request from *A* as $c_{1}$ has shared to another device.

### 5.3. Optimizing PKI Usage in Decentralized CFA Schema

In our solution, devices can verify each other mutually while defending the attestation process from malicious nodes. Similar to other research, mitigating impersonation and session-hijack, we use PKI to keep the verification confidential and integrated. Moreover, the usage frequency of public-key could be reduced in every verification process after the deployment of our secret slice mechanism.

Our motivation is declared as follows: (i) To identify which device sends the challenge, the verifier uses its private key to sign the message digest. Using a private-key alone does not guarantee confidentiality as every node holds its public key. (ii) Likewise, if we use the private key of a verifier to identify a challenge and use symmetric encryption to keep it confidential. It is hard for this approach to defend *response defraud* from a malicious verifier. The reason has been declared in our threat model. (iii) We deploy secret slices to mitigate such threats under the protection of symmetric keys. Therefore, the shared slices already determine the identity of generated challenges. Before challenges are exhausted, using the private key (for identification) in every attestation process is unnecessary. This means the usage of PKI could be concentrated into a smaller procedure. Thus, embedded devices can lower their computational burden to a certain extent in each verification session.

We elaborated a *registration phase* to protect the secret delivery in our verification schema. It works as follows: (i) We use a temporary master node for the key exchange, in line with public-key infrastructure; (ii) The secret slicer generates the matrix of test cases and builds the secret by recording the corresponding control flow with the test cases. For example, the secret slice that passed from *B* to *A* is $Secret_{BA}$. After generating secret slices, node *B* only keeps the challenge name and drops the response; (iii) The node delivers secret slices to its peer. This involves digesting the secret slices, signing the message digest with its private key, and encrypting the secret slices using their session key. Then, the temporary master node is withdrawn from the cluster.

## 6. System Implementation

**Figure 8:**
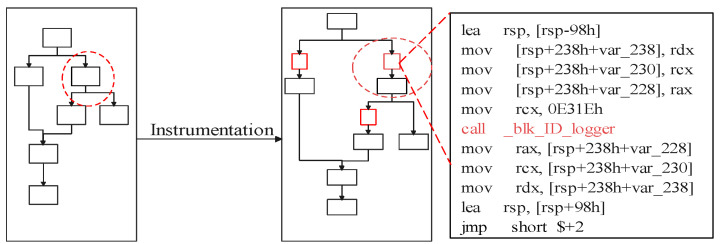
Instrumentation.

In this section, we describe our implementation of CFRV on Raspberry-Pi 3b with the OP-TEE. The Raspberry-Pi series is a popular platform in the embedded devices’ ecosystem. We use *ARM Trustzone* as the trust anchor, which isolates *secret slices* and *CF-logger* from target software by hardware support of processors. As ARM takes a major part of the MCUs market, it is reachable for CFRV to be transplanted into real-world usage like automotive and smart homes. We use the LLVM [28] as our compile chain for the instrumentation (the Figure 8).

Note that, when a function is repeatedly executed, the performance impact brings by interacting with the control-flow recording module seems unacceptable [9]. To deal with, in line with instrumentation-based schema (like C-FLAT), we use *loop-counter* instead of stamping inside the loops. In this part, we elaborate on our complete attestation schema in the following three phases.

### 6.1. Initialization

The initialization phase is to set up the attestation’s basic requirements. It includes the compilation of target software, the *instrumentation*, and the generation of secret slices. In this phase, devices are secure, as the security assumption declared that this process happens only once on the temporally existing master node.

(i) In order to deploy an instrumentation process for identifying each control flow, CFRV uses *Clang* to compile source code into its intermediate representation (IR). Instrumentation is a way to statically make stamps on the control flow by inserting identifiers between transfer instructions in the IR. These stamped instructions (the block-id and jump to the trampoline) are used for recording its execution path. In Figure 8, the block-id inserted into the IR is a 16-bit random number. Rather than instrumenting before every block, CFRV reduces the instrumentation density as long as the control flow record is unique. Furthermore, we use a *loop-counter* instead of stamping inside the loops. Then, we build these instrumented IRs into binaries through the LLVM backend.
(ii) The secret slicer transforms random seeds to build a set of *challenge–response pairs* by covering execution paths automatically. In our proof-of-concept, we built fuzzer-like test-cases generator based on the AFL [29]. For example, changing the precision of floating point variables can generate different test cases. Moreover, to ensure every slice is totally different from others, the number of devices (*n*) cannot be too large. In our implementations, we regard 8 as a normal threshold of *n* and the size of each secret slice is 15 kb.
(iii) The master node delivers unique binaries along with their certificates (public-key pair) to other devices. After that, it would be excluded from the cluster as we cannot always ensure its safety.

### 6.2. Registration

In this phase, peer devices exchange their secret slice under the protection of public-key infrastructure.

(iv) Devices slice their generated test-cases into disjoint parts based on the number of other devices (*N*) with a constraint that the functionalities for each slice are the same. For example, the content of $Secret_{BA}$ is totally different from $Secret_{BC}$ as a certain test case could and could only be picked once (Equation (1)), while the control-flow they trigger are the same (Equation (3)). After sharing secret slices, the node *B* only keeps its challenge as *challenge_slices* and drops the response in the secret slice.
(v) The node delivers its secret slices under the protection of PKI that deploys in (iii). For example, device *B* sends *A* the message which contains: $Sign[K_{PriB}:Hash\left(Secret_{BA}\right)]$, $Enc[K_{BA}:Secret_{BA}],$ $Enc[K_{PubA}:K_{BA}].$

This way, devices can securely share their secret slices and session key.

### 6.3. Verification

**Figure 9:**
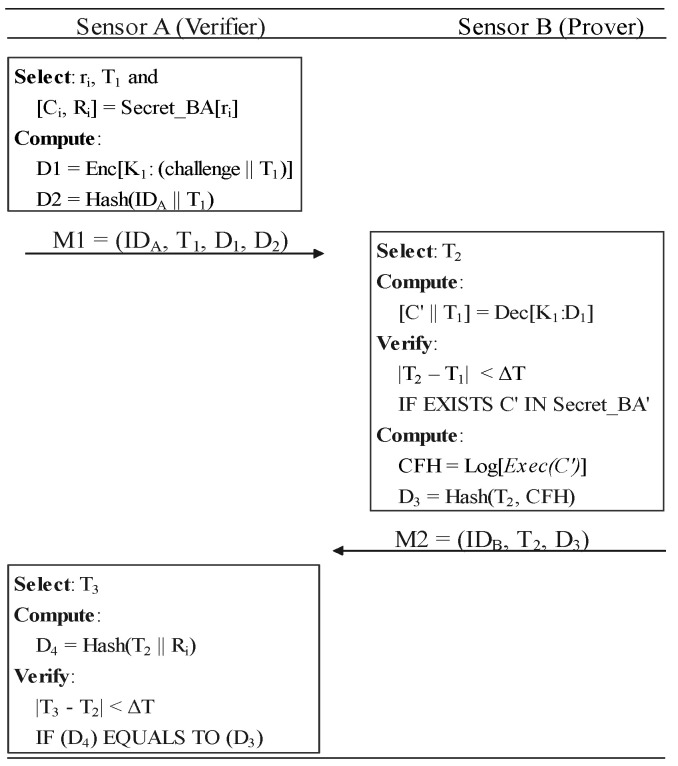
The verification phase.

**Figure 10:**
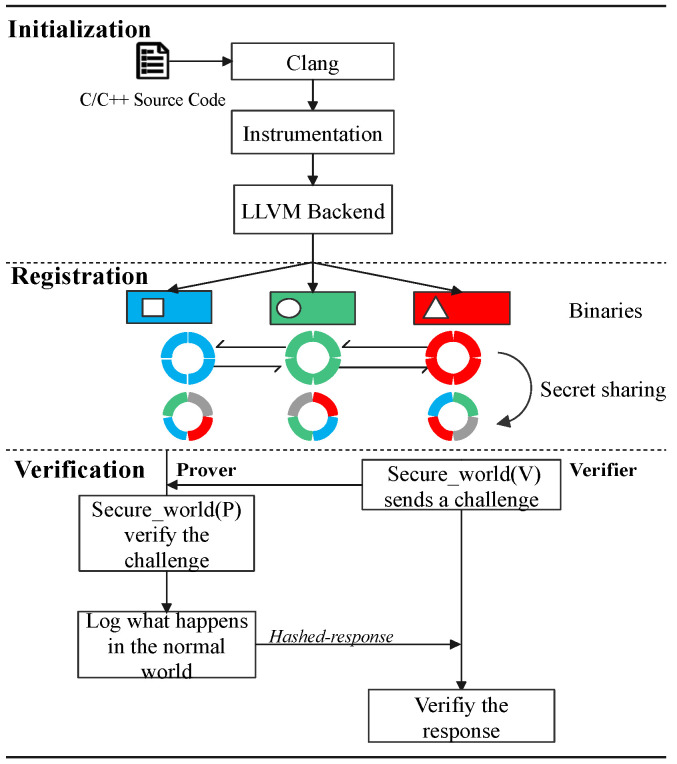
Relationship among components in CFRV.

The verification process allows one sensor node (the verifier) to determine the run-time integrity of certain software on other nodes (the prover). It consists of three steps shown in Figure 9, which corresponds to the *verification phrase* in Figure 10. Note that, without secret slices, using symmetric encryption alone is vulnerable to *response fraud* that we declared in Section 3.

(vi) The verifier generates a time-stamp $T_{1}$ and selects a random number $r_{1}$. Verifier uses $r_{1}$ to pick a *Challenge–Response Pair (CRP)*$\left[c,R\right]=Secret_{BA}\left[r_{1}\right]$ from secret slice which is sent by the prover. The verifier computes $D_{1}$ and $D_{2}$. $D_{1}$ is encrypted with their symmetric key to secure the random challenge *c*. Then, it sends $M_{1}$ as a verification request to B.
(vii) The prover generates a time-stamp $T_{2}$ and decrypt $T_{1}$ and challenge *c* from $D_{1}$. We use $|T_{2}-T_{1}|<\Delta T$ to reject the time-out messages. The prover ensures that *c* is actually from $Secret_{BA}$ shared in the registration phase. The prover uses the corresponding software to execute challenge, *c*, after admitting it is from $Secret_{BA}$. CFH is a control-flow record consisting of the hashes of basic block-id consequences. Then, the prover sends $M_{2}$ to the verifier along with its loop metadata.
(viii) Verifier generates a time-stamp $T_{3}$ and computes $D_{4}=Hash\left(T_{2}\|R\right)$. If $|T_{3}-T_{2}|<\Delta T$ and $D_{4}==D_{3}$, the verifier would accept prover’s integrity based on the control-flow records which matches the response stored in $Secret_{BA}$. $D_{4}==D_{3}$ means that corresponding control-flow is actually integrated.

## 7. Evaluations

In this section, we examined the performance of CFRV by analyzing our counter-measure to the research questions and the computational cost it brings.

### 7.1. Safety

We evaluate the safety of the verification process in related mechanisms according to our security assumptions. In Table 2, we list several common threats along with our research question for evaluating decentralized approaches to control-flow attestation.

#### 7.1.1. Impersonation

Regardless of its performance burden, public-key usage in decentralized attestation schema could mitigate the impersonation well. Challenges are required to be signed, even in centralized approaches; otherwise, an impersonated verifier can compromise it. In Table 2, the semi-circle means the verifier has not signed the challenge, or it has not declared in the scheme. In CFRV, the challenge is uniquely specified in the secret slices to identify each node. An unexpected challenge could not be accepted.

#### 7.1.2. Session Hijack

In CFRV, the challenge is uniquely specified in the secret slices to identify each node. An unexpected challenge could not be accepted. If a scheme transfers challenges in plaintext, adversaries can modify the message or replace the payload with a poisoned challenge even if the real verifier is trustworthy. In Table 2, semi-circles mean the response is not encrypted by the prover or has not been declared in the scheme. The communications between peer devices in CFRV are under the protection of symmetric encryption. Furthermore, it uses a one-time challenge and time-stamp to defend against the replay attack.

#### 7.1.3. Poisoned Challenges

In decentralized approaches, a malicious device can also become a verifier in the peer nodes. As we declared in research question 1, a decentralized CFA schema should mitigate the *poisoned challenges* if the prover is required to execute challenges (to formulate a proof-of-execution) in the decentralized schema. We build an attack vector as the payload of verification with return-oriented programming. Our experiment shows that the software running on a prover was compromised after executing the challenge as its CFA schema did not check the challenge. In centralized approaches, the adversary can realize *poisoned challenge* by the session hijack. It uses an attack vector to replace the challenge if the verification request is not encrypted.

In CFRV, the prover can refuse a verification request under the protection of *secure world (P)* if the challenge has not been shared before. The adversary cannot replace a legalized challenge with a poisoned one as their confidentiality is under the protection of symmetric encryption.

#### 7.1.4. Response Defraud

With reverse engineering, a malicious node can launch the *response defraud* if it obtains the instrumentation detail and software structure (the CFG). In most schemes, adversaries can satisfy such requirements because instrumentation and software are stored in the *normal world* (or are more weakly protected). Furthermore, instead of colliding hash results or basic-block identifiers, enumerating the execution path of the target software would be more achievable.

As declared in the research question 2, we examined the *response defraud* in a decentralized schema with the following steps to bypass control flow attestations. In our experiment, the software being verified is identical. Firstly, the adversary knows which control flow the challenge triggers. Moreover, it gets the instrumentation detail from the *normal world* of the victim. The malicious node attempts to enumerate the execution path by sequencing hashes of block-ids compared with its response. Without instrumentation inside loops, the complexity of a successful enumeration is no harder than finding a specific sub-graph in a DAG (directed acyclic graph). Then, the adversary forges a block-id sequence in the enumerated execution path. Finally, it scrabbles up its hashes’ results to complete the *response defraud*. In centralized approaches, *response defraud* can be realized in the way of impersonation if the verifier has not signed the verification request.

To extinguish the *response defraud*, CFRV enforces that challenges in one verification process cannot be used with other devices. Once the adversary forwards a challenge to defraud a response, the prover would reject this request; this is because the forwarded challenge does not exist in secret slices used for another session.

### 7.2. Performance

#### 7.2.1. Protocol Efficiency

We evaluate the verification duration of our schema to examine the effect of optimizing public-key usage. The specific cryptographic algorithm chosen by each scheme is not entirely the same (e.g., the public-key algorithm or the hash functions). In order to illustrate the efficiency difference caused by the design of the verification protocol, we use *openssl* (https://www.openssl.org/accessed on 6 July 2022) to unify the cryptographic operation in the evaluations.

In our experiment, the timer activates when a verifier builds a challenge and ends after the response is verified. The construction and execution time of challenges is excluded based on our criterion.

**Figure 11:**
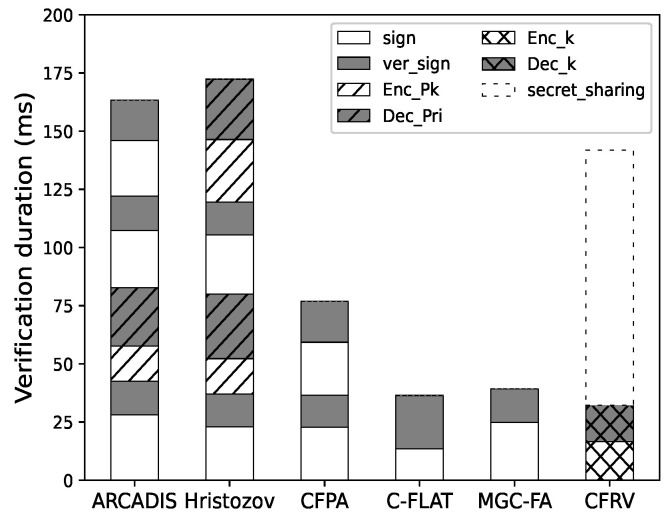
One round of attestation.

**Figure 12:**
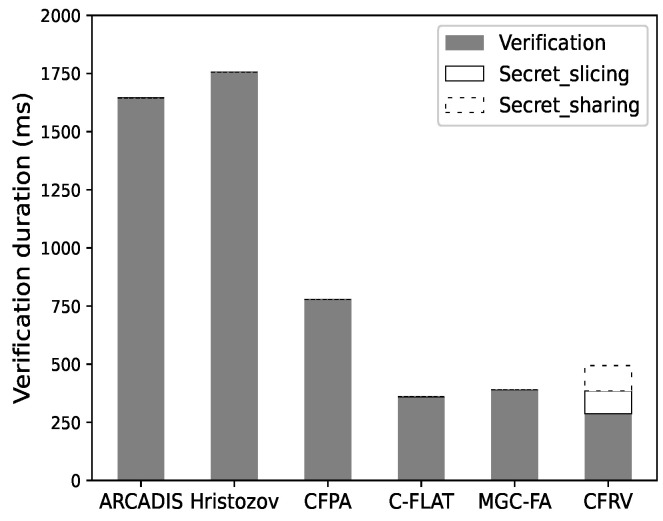
Ten-round attestation.

Figure 11 shows that it is not easy for CFRV to have a clear advantage in a single-pass verification as it takes additional time to share secret slices. If the verification repeats more than once, as shown in Figure 12, CFRV could reveal its advantage because of the public-key usage optimization. The cryptographic operation’s duration is reduced by 40% compared to related decentralized schemes. It shows that the secret slices could concentrate public-key usage into a single stage rather than using it in every verification. In this case, this could be a better solution in control-flow attestation of embedded systems.

#### 7.2.2. Overhead

We use RIPE [30] as the benchmark and turn off the ASLR and DEP protection. In the evaluations, we conducted 100 successful exploits on the vulnerable program. CFRV detects no less than 98 of 100 available attacks in the default instrumentation density (30% specified in this program). The remaining two attacks were also detected after we increased the instrumentation density. Furthermore, in this part, we evaluate the capability that CFRV defends against the control flow hijack and the target software’s performance burden brought by this security mechanism. In Table 3, *firstROP.c CallARG.c* (https://github.com/nalamchaitanya/ROP accessed on 6 July 2022) are two software programs commonly used for demonstrating ROP attacks. The *Encrypt.cpp*, *LED-controller.cpp*, *MotionDriver.cpp* are programs designed for embedded devices, which we set a *buffer overflow* in their functions as the vulnerability. Its overhead, the extra running duration of instrumented programs, is calculated as Equation (4):

(4) $$Overhead=\frac{T_{inst}-T_{orig}}{T_{orig}}\times 100\%$$

In Table 3, the experiment shows that the overhead brings by instrumentation ranges from 1.2% to 21.8% according to its density. In other words, compared to the cryptographic program, functions for embedded devices are simpler in structure and require less extra code to be inserted. In this condition, instructions used for interacting *normal world* with *secure world* account for a less proportion, so they have a relatively weaker impact on the execution time of embedded programs. Moreover, due to the optimization of the public-key usage in the CFRV, its overall performance cost is still acceptable in the process of not real-time or high-frequency verification.

## 8. Conclusions

This paper patched the decentralized CFA schema with a secret slice mechanism to make the prover able to distinguish the origin of a request. As a result, it can mitigate the *response defraud* and make a kind of POX (that regards input as challenges) suitable for the decentralized CFA schema. Moreover, CFRV detects no less than 98% of the code-reuse attack without a trusted center under a low instrumentation density. In this case, CFRV is more suitable for the decentralized scenario that requires the remote device to provide a trustful result. We also investigated how to reduce the cryptographic operations of decentralized CFA. Our evaluations show that limiting the PKI to a crucial stage lowers the private key usage in every challenge–response round. The cryptographic duration was therefore reduced by 40% compared to related decentralized schemes. Nevertheless, concentrating the PKI usage in the *registration* step constrains the network to a single boot-up operation. No new node can enter without using a trusted node to restart the process. Extending the flexibility of this schema will be studied in our future work. As an instrumentation-based schema, its overhead is supposed to be further reduced in future works (e.g., to integrate a specialized hardware module to log the control flow events). Moreover, each node in this schema needs to store and exchange the secret slices. The space of software input cannot be too large. Furthermore, as the trust-anchor, CFRV relies on the *ARM Trustzone* to provide its security feature. It is also important to harden the *Trustzone* itself in the future.

## Figures and Tables

**Table 1:** Symbol summary.

Symbol	Description	Procedure	Description
$C_{i}$	Challenge slice	Exec(c)	Execute the input *c*
$R_{i}$	Response slice	$Sign(K_{Pri}:m)$	Sign *m* using private key $K_{Pri}$
*c*	a challenge	$Verify(K_{Pub}:m)$	Verify *m* using public key $K_{Pub}$
$Secret_{CA}$	Secret slice from C to A	$\left[r_{i}\right]$	Generate a random number
*T*	Time stamp	Enc(K:m)	Encrypt *m* using symmetric key *K*
*n*	The number of devices	Dec(K:m)	Decrypt *m* using symmetric key *K*
*S*	Software input	Hash(A||B)	Cryptographic hash function

**Table 2:** Safety analysis of related schemes.

Scheme	Single-Point Failure	Impersonation	Session Hijack	Poisoned Challenge	Proof of eXecution	Response Defraud
C-FLAT [9]	✕	◐	◐	-	✕	-
Lo-FAT [20]	✕	◐	◐	-	✔	-
Tiny-CFA [16]	✕	✔	◐	-	✔	-
ScaRR [17]	✕	◐	◐	-	✔	-
CFPA [23]	✔	✔	◐	✔	✕	✕
Hristozov et al. [24]	✔	✔	✔	✔	✕	✕
ARCADIS [25]	✔	✔	✔	✔	✕	✕
CFRV	✔	✔	✔	✔	✔	✔

Item ‘-’ means this approach was not applicable. Item ‘✔’ means this threat can be mitigated.

**Table 3:** Performance.

Program	Code Injection	JOP	ROP	Executable Size	Overhead
firstROP.c	✔	-	✔	8.7 kb	1.5%
CallARG.c	✔	✔	✔	8.6 kb	1.2%
Encrypt.cpp	✔	✔	✔	29.8 kb	21.8%
LEDcontroller.cpp	✔	✔	✔	18 kb	7.2%
MotionDriver.cpp	✔	✔	✔	17.3 kb	16.0%

Item ‘-’ means this approach was not evaluated. Item ‘✔’ means this approach can be detected.

## Data Availability

Not applicable.
